# Silibinin Ameliorates *O*-GlcNAcylation and Inflammation in a Mouse Model of Nonalcoholic Steatohepatitis

**DOI:** 10.3390/ijms19082165

**Published:** 2018-07-24

**Authors:** Su Jin Lee, Min Jung Nam, Da Eun Lee, Jeen-Woo Park, Beom Sik Kang, Dong-Seok Lee, Hyun-Shik Lee, Oh-Shin Kwon

**Affiliations:** School of Life Science, College of Natural Science, Kyungpook National University, Daegu 41566, Korea; neojove79@naver.com (S.J.L.); nmj0604@naver.com (M.J.N.); Starkr0@hanmail.net (D.E.L.); parkjw@knu.ac.kr (J.-W.P.); bskang2@knu.ac.kr (B.S.K.); lee1@knu.ac.kr (D.-S.L.); leeh@knu.ac.kr (H.-S.L.)

**Keywords:** silibinin, *O*-GlcNAcylation, non-alcoholic steatohepatitis, proteomics, inflammation

## Abstract

The mechanisms underlying the progression to non-alcoholic steatohepatitis (NASH) remain to be elucidated. In the present study, we aimed to identify the proteins involved in the pathogenesis of liver tissue inflammation and to investigate the effects of silibinin, a natural polyphenolic flavonoid, on steatohepatitis. We performed comparative proteomic analysis using methionine and choline-deficient (MCD) diet-induced NASH model mice. Eighteen proteins were identified from the two-dimensional proteomic analysis, which are not only differentially expressed, but also significantly improved, by silibinin treatment. Interestingly, seven of these proteins, including keratin cytoskeletal 8 and 18, peroxiredoxin-4, and protein disulfide isomerase, are known to undergo GlcNAcylation modification, most of which are related to structural and stress-related proteins in NASH model animals. Thus, we primarily focused on how the GlcNAc modification of these proteins is involved in the progression to NASH. Remarkably, silibinin treatment alleviates the severity of hepatic inflammation along with *O*-GlcNAcylation in steatohepatitis. In particular, the reduction of inflammation by silibinin is due to the inhibition of the *O*-GlcNAcylation-dependent NF-κB-signaling pathway. Therefore, silibinin is a promising therapeutic agent for hyper-*O*-GlcNAcylation as well as NASH.

## 1. Introduction

Nonalcoholic fatty liver disease (NAFLD) includes two pathologically clear conditions with different recuperation: hepatic fat accumulation (steatosis) and non-alcoholic steatohepatitis (NASH). For the diagnosis and treatment of NAFLD, it is important to comprehensively assess pathological factors according to a systematic guideline [1,2]. Non-alcoholic steatohepatitis (NASH) is closely related to the components of metabolic syndrome, namely, obesity, insulin resistance, and dyslipidemia. Little is known about the causes of transition from simple steatosis to NASH. However, the lipid molecules accumulated in NASH appear to play a direct role in hepatocellular injury and inflammation. Accumulated molecules in hepatic cells are metabolized through mitochondrial and peroxisomal oxidation, which increases reactive oxygen species (ROS) production [3,4]. ROS are considered to induce the production of pro-inflammatory cytokines such as tumor necrosis factor (TNF)-α, leading to cell necrosis and apoptosis. Moreover, the proliferation and activation of hepatic stellate cells (HSCs) can be directly induced by ROS. Toll-like receptors (TLRs) might be the key receptors regulating inflammation in the liver, myofibroblast accumulation, and fibrosis. LPS-mediated TLR4 activation plays a crucial role in fibrogenic signaling pathways and HSC activation [5].

Milk thistle (*Silybum marianum*) has been used for centuries to treat liver disorders. Silibinin is a major active constituent of silymarin, an extract obtained from milk thistle fruits and seeds [6]. Various studies demonstrated that silibinin not only exhibits anti-carcinogenic effects, but also exerts a number of additional biological effects such as antioxidant, pro-apoptotic, and anti-inflammatory effects [7,8,9]. It is clear that silibinin is effective in preventing severe oxidative stress and preserving hepatic mitochondrial bioenergetics in NASH. However, the mechanisms underlying the effects of silibinin on NASH have not been fully elucidated.

The hexosamine biosynthetic pathway (HBP) regulates the post-translational modification of cytoplasmic and nuclear proteins by *O*-linked β-*N*-acetylglucosamine (*O*-GlcNAc) [10]. *O*-GlcNAc is thought to play an important role in almost all aspects of cellular functioning. *O*-GlcNAcylation is highly dynamic through the action of two intracellular enzymes, namely, *O*-GlcNAc transferase (OGT) and *O*-GlcNAcase (OGA) [11]. OGT utilizes UDP-GlcNAc, the end product of HBP, to transfer *O*-GlcNAc group into serine or threonine residues on the target protein, while OGA catalyzes the reverse reaction to remove the sugar molecule [12,13]. So far, thousands of cytoplasmic and nuclear proteins *O*-GlcNAcylated by OGT have been identified [14]. The level of O-GlcNAcylation is considered a nutrient sensor because it is modulated by the availability of glucose, fatty acids, amino acids, and nucleotides. Abnormal *O*-GlcNAcylation is associated with many human diseases such as diabetes, neurodegenerative disorders, cardiac disease, and cancer. Upregulation of *O*-GlcNAcylation is a common feature of many types of cancer, including breast and prostate cancer, but the role in inflammation progression remains unknown.

Few studies have reported the use of proteomics to study the effects of silibinin in NASH. Thus, in the present study, we attempted to characterize the hepatic proteome by using methionine and choline-deficient (MCD)-diet-fed mice, and then we analyzed the effect of silibinin on steatohepatitis. The administration of the MCD diet to mice has been proven to be a useful experimental model for steatohepatitis [15,16]. The MCD diet induces oxidative stress as well as steatosis, fibrosis, and inflammation, which seem to be important for the progression to steatohepatitis. Here, with particular attention to the involvement of *O*-GlcNAcylation in MCD-induced steatohepatitis, we report the search for differentially expressed proteins in inflammation and the effect of silibinin treatment.

## 2. Materials and Methods

### 2.1. Animals

C57BL/6J male mice were purchased from Chungang Science (Daegu, Korea). We normally used 7–10 number mice (seven weeks old) for each experiment. All mice were conditioned to proper temperature (20–24 °C) and proper humidity (45–55%) for a week. Mice were classified into three groups. The control group (*n* = 10) was fed the normal diet for three weeks. The second group (*n* = 8) was fed the MCD diet and injected with lipopolysaccharide (LPS; Sigma Aldrich Chemicals, St. Louis, MO, USA) for three weeks. The third group (*n* = 7) was fed the MCD diet with LPS as well as treated with silibinin (Sigma Aldrich Chemicals) for three weeks. LPS was intraperitoneally injected once a week (1 mg/kg), whereas silibinin was injected daily (1 mg/kg).

### 2.2. Two-Dimensional Gel Electrophoresis

Two-dimensional gel electrophoresis (2DE) was performed using an established procedure [17]. Briefly, mouse liver samples were lysed with urea buffer. Each liver-lysed sample was loaded onto a 24-cm immobilized linear gradient strip (pH 4–7; GE Healthcare Life Science, MA, USA, and then rehydrated in an Ettan IPGphor isoelectric focusing (IEF) system. For the second dimension, the IPG strips were incubated in an equilibration buffer [125 mM Tris (pH 6.8) containing 6 M urea, 30% glycerol, and 2% SDS] containing 65 mM DTT for 15 min, and then incubated for 15 min in an equilibration buffer supplemented with 2.5% (*w*/*v*) iodoacetamide. The strips were transferred onto a 12% gel for SDS-PAGE to separate proteins by MW. Gel staining with Coomassie Blue was performed as described by Kang et al. [18].

### 2.3. In-Gel Digestion and MALDI-TOF Analysis

Protein spots were extracted from the gels, and in-gel digested with trypsin. Then, the samples were prepared for MALDI-TOF protein analysis. Acetonitrile (ACN; 50%) was used to destain Coomassie Blue-stained gel spots, and 0.1 M ammonium bicarbonate was used to eliminate salts. The pellets were dried using a speed vacuum and treated with trypsin (50 mM NH_4_HCO/20 μg/mL trypsin) at 37 °C overnight. The dried pellets were dissolved in 0.5% trifluoroacetic acid (TFA) and desalted using C18ZipTip (Millipore, Billerica, MA, USA) according to the manufacturer’s protocol prior to loading on the steel MALDI plate. For MALDI-TOF analyzed using Explorer software (Applied Biosystems, Waltham, MA, USA) and MS-fit program by using mouse protein sequences in the NCBI non-redundant database as a reference set. The search parameters included a precursor mass tolerance of 50 ppm peptide mass tolerance and one maximum missed cleavage. In addition, up to two missed cleavages were allowed; oxidation of methionine was set as a variable modification, and carboxyamidomethylation of cysteine as fixed modification.

### 2.4. Immunoblotting

Immunoblotting was used to validate the differential expression of proteins identified by MS. Samples were lysed with a lysis buffer containing 50 mM HEPES pH 7.4, 150 mM NaCl, 1 mM EDTA, 2 mM sodium orthovanadate, 1% NP40 and 1 μM okadaic acid prior to protein quantification with Coomassie Protein Assay Reagent (BioRad, Hercules, CA, USA). About 50 μg of each samples were diluted in the Laemmli sample buffer (final concentration: 50 mM Tris pH 6.8, 2% SDS (*w*/*v*), 10% (*v*/*v*) glycerol 0.01% (*w*/*v*) bromophenol blue) and separated by 1D-SDS-PAGE following the standard procedure. After electroblotting, the separated proteins were transferred onto nitrocellulose membranes (GE Healthcare Life Science, MA, USA), and the membranes were blocked with 5% *w*/*v* skim milk in TBST [50 mM Tris pH 8.0, 150 mM NaCl, and 0.1% Tween-20 (*v*/*v*)] for 30 min. The membranes were then incubated in the primary antibody solution prepared in TBST containing 0.02% (*w*/*v*) sodium azide for 2 h. Then, the membranes were washed in TBST (3 × 10 min) and probed with the appropriate horseradish peroxidase-coupled secondary antibody (Cell Signaling Technology, Danvers, MA, USA). Antibodies against α-SMA (1:1000; Epitomics, Burlingame, CA, USA), Cytokeratin 8 (CK8), Cytokeratin 18 (CK18), AnnexinA5, regucalcin, OGT, β-*O*-linked *N*-acetylglucosamine, Prx4, and P65 (1:1000; Abcam, Cambridge, MA, USA) were used as probes. The anti-GAPDH antibody (1:1000; Santa Cruz, CA, USA) was used as a loading control.

### 2.5. Immunohistochemistry

The livers were extracted from mice, and the slide sections were stained with hematoxylin andeosin (H&E) for histological analysis. Other slide sections from the paraffin block of liver tissue were stained with primary antibodies for protein localization. Sample sections were reacted with the appropriate secondary antibodies. The immunohistochemical reactions were visualized using 3,3-diaminobenzidine (DAB) reagent.

### 2.6. Immunoprecipitation

Liver and Raw 264.7 cells were homogenized in NP40 buffer containing protease inhibitors. Lysates were incubated overnight with 10 μg of either anti–P65 (Santa Cruz Biotechnology, CA, USA) and β-*O*-linked *N*-acetylglucosamine, followed by the addition of 200 μL Dynabeads-Protein-A (Thermo Fisher Scientific, Waltham, MA, USA) conjugated slurry and incubation at 4 °C for 2 h. The immunoprecipitates were collected and washed three times with NP40 buffer.

### 2.7. Quantitative Reverse-Transcription Polymerase Chain Reaction

Quantitative real-time (RT) PCR analysis was performed using an established procedure [19]. Total RNA was extracted using QIAGEN extraction kit and reverse transcription was performed using SuperScript III RNase H (Invitrogen, Waltham, MA, USA). Quantitative RT-PCR was performed using SYBR Green I and a LightCycler (Roche Diagnostics, Castle Hill, Australia). For normalized gene expression, 18S was used as the reference gene in all experiments. Table S1 lists the primers used for RT-PCR analysis.

### 2.8. Statistical Analysis

All data are expressed as mean ± SD. Statistical analysis was performed using nonparametric Mann–Whitney tests. For all experiments, *n* = 7–10 animals per group. Significance was established using Mann–Whitney tests, with significance *p* < 0.05, corrected for multiple comparisons.

## 3. Results

### 3.1. Effect of Silibinin on MCD Diet-Induced Steatohepatitis

**Figure 1:**
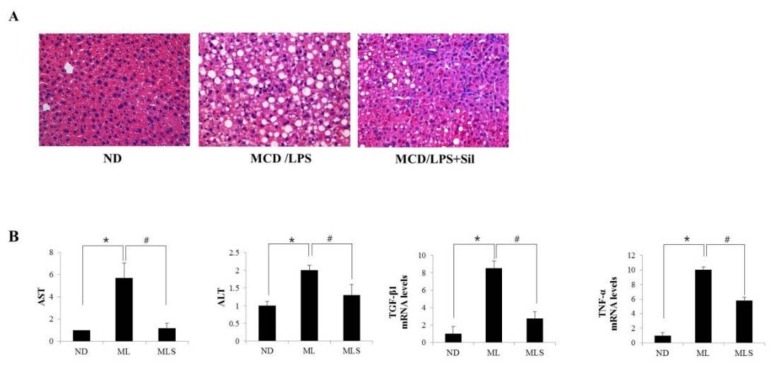

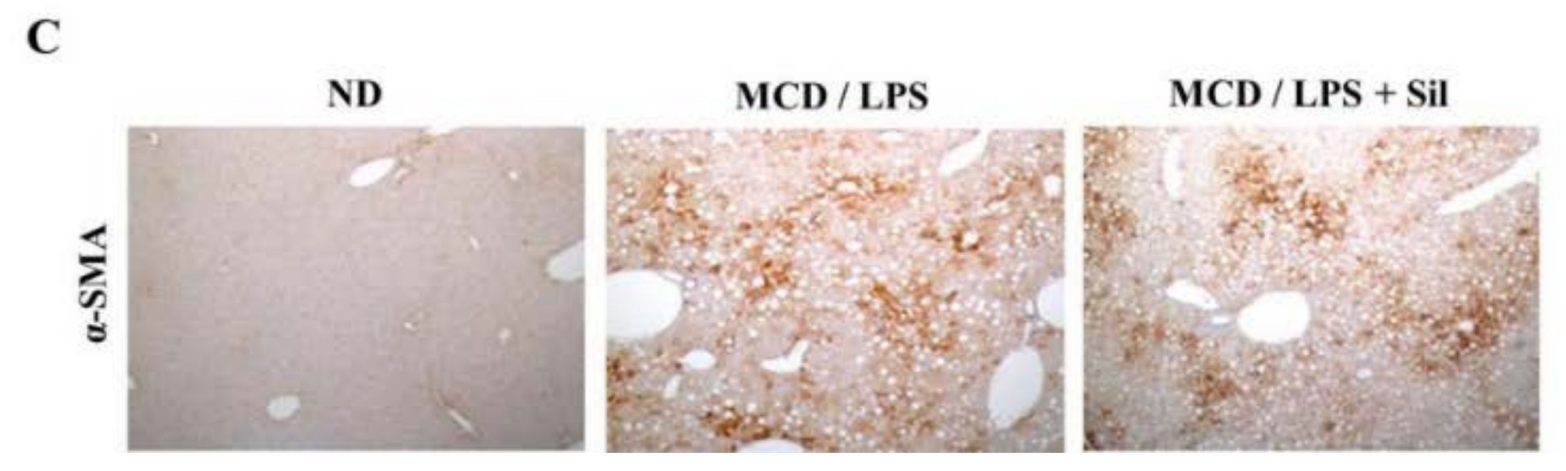
Effect of silibinin on hepatocellular injury. Mice were fed ND, MCD/LPS diet, or MCD/LPS diet + silibinin for three weeks. (**A**) The histology of the liver sections from these three groups was assessed by hematoxylin-eosin (H&E) staining (original magnification, 200×). (**B**) Serum AST, ALT values and the expression levels of the proteins involved in the development of steatohepatitis, such as *TGFβ1*, *TNF-α*, *IL6*, and *iNOS*, were measured by qRT-PCR. Genes were normalized to GAPDH RNA as an internal standard. Fold-increase data are expressed. Data are the mean ± SD (*n* = 7/group). * *p* < 0.05 vs. ND group, and # *p* < 0.05 vs. MCD/LPS group. (**C**) Liver sections of the mice fed ND, MCD/LPS, or MCD/LPS + silibinin were subjected to immunohistochemical analysis for α-SMA (original magnification, 100×).

MCD diet-fed mice showed hepatic histological findings similar to those observed in the human cases of NASH [20]. Administration of LPS to MCD mice induces higher inflammatory activity, compared to the mice fed only MCD. As shown in Figure 1A, injecting LPS into MCD-fed mice for three weeks resulted in a significant increase in lipid accumulation, whereas treatment with silibinin ameliorated liver pathology. Silibinin is a potential antioxidant and anti-inflammatory agent [21,22]. The liver–body weight ratio was significantly decreased in the MCD/LPS-fed mice group (Figure S1). The levels of serum AST and ALT increased in MCD/LPS-fed mice, but dramatically decreased in silibinin-treated mice, as in ND mice (Figure 1B). To explore the levels of proteins involved in the development of steatohepatitis, we performed qRT-PCR analysis of the liver homogenate. The mRNA levels of cytokines, TGF-β, and TNF-α increased in the liver of the MCD/LPS group, but significantly decreased in the group treated with silibinin. During the progression of steatohepatitis, the increase in the levels of pro-inflammatory cytokines could induce α-SMA, a unique marker for activated hepatic stellate cells and myofibroblasts. Thus, immunohistochemical analysis was performed using antibodies against α-SMA (Figure 1C). α-SMA levels significantly increased in LPS-injected MCD mice but decreased in silibinin-treated mice. Taken together, these results indicated that silibinin alleviates high-fat-induced hepatic steatosis and inflammation in MCD/LPS-fed mice.

### 3.2. DE Analysis for Differentially Expressed Proteins

The proteomes of the mouse liver supplemented with MCD/LPS diet or silibinin-treated MCD/LPS diet were analyzed using 2DE. Supporting information (Figure S2) shows a typical 2DE pattern of the mouse liver. Protein spots significantly affected by MCD/LPS diet and silibinin treatment are marked by arrows with numbers. Protein spots from the preparative gel were subjected to in-gel tryptic digestion and MALDI–TOF MS analysis. Table 1 lists the differentially expressed proteins, most of which exhibit a minimum of 1.5-fold difference in expression. About 18 differentially expressed proteins were grouped and classified according to their biological function. Majority of the differentially expressed proteins are related to metabolism and structural and genomic processes. In addition, the levels of silibinin-treated mice significantly improved. Thus, this result suggests that silibinin is involved in ameliorating the disturbance of metabolic homeostasis and hepatocellular stress in steatohepatitis. In particular, the seven proteins shown in blue in Table 1 are deemed most interesting because they can be modified by *O*-GlcNAcylation.

### 3.3. Suppression of O-GlcNAcylation by Silibinin in Steatohepatitis

**Figure 2:**
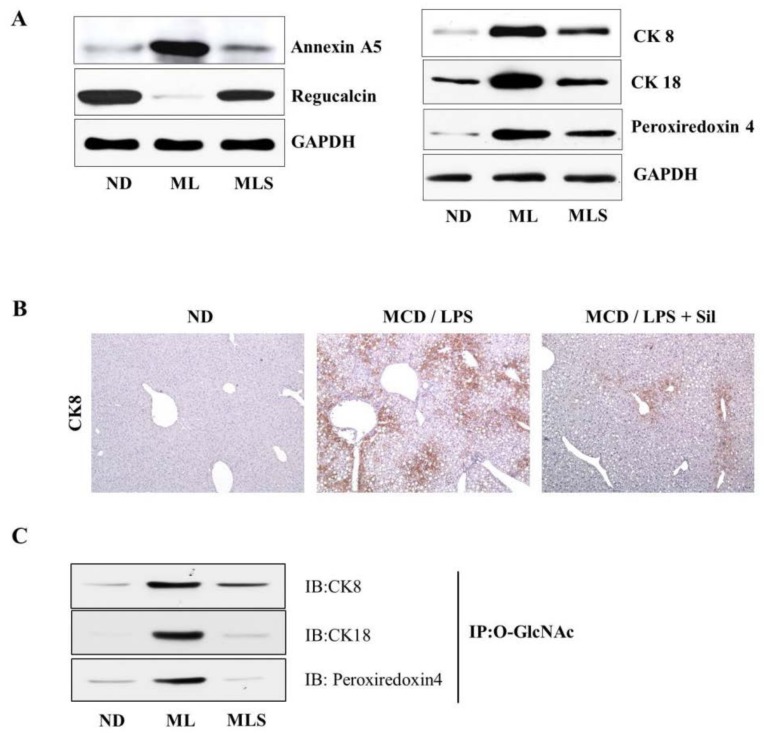
Validation of the differentially expressed protein. Liver tissue extracts were prepared from the mice fed ND, MCD/LPS, or MCD/LPS + silibinin. (**A**) The left panel shows the hepatic expression levels of annexin A5 and regucalcin, as determined by immunoblotting using equivalent amounts of total liver protein. The expression levels were normalized relative to GAPDH. The right panel shows the relative expression levels of CK8, CK18, and PRX4. Data represent three independent experiments. (**B**) Liver sections were subjected to immunohistochemical analysis using antibodies to CK8. ×100 magnification. (**C**) *O*-GlcNAc was immunoprecipitated with anti-*O*-GlcNAc antibody, and then immunoblotted against CK8, CK18, or peroxiredoxin-4, respectively.

To validate our findings from the proteomics approach, we performed immunoblotting and immunohistochemical analyses to evaluate the changes in protein levels in steatohepatitis or after silibinin treatment. As shown in Figure 2A (left panel), we demonstrated by immunoblotting that annexin A5 and regucalcin are significantly upregulated and downregulated in MCD/LPS-fed mice, respectively. However, the effects on protein expression significantly improved in silibinin-treated mice as well as in ND mice, which are consistent with the results of proteomic analysis. Similarly, we confirmed that the expression of keratin cytoskeletal 8 (CK8) and keratin cytoskeletal 18 (CK18), and peroxiredoxin-4 (PRX4) was dramatically upregulated in the MCD/LPS-fed mice, as shown in the right panel, but ameliorated in silibinin-treated mice. The findings of immunoblotting of CK8 protein were further confirmed by immunohistochemical analysis (Figure 2B). In addition, among the proteins tested in this study, CK8, CK18, and PRX4 were of particular interest because these proteins can be modified by *O*-GlcNAcylation. Thus, we further tested the levels of *O*-GlcNAc modification of these proteins by using immunoprecipitation and immunoblotting analysis (Figure 2C). Indeed, we observed a dramatic increase in the level of *O*-GlcNAc modification of all proteins in MCD/LPS-fed mice, which seems consistent with the results listed in Table 1.

**Figure 3:**
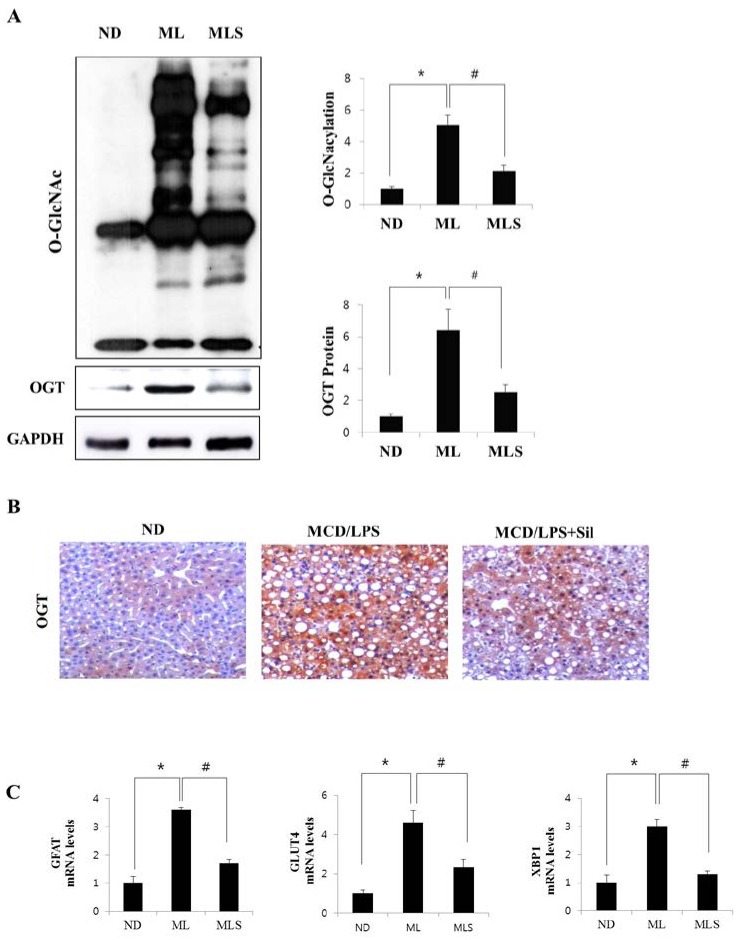
*O*-GlcNAcylation and the expression levels of the proteins associated with the HBP pathway. (**A**) The upper panel shows the hepatic expression levels of *O*-GlcNAcylation in ND, MCD/LPS, or MCD/LPS + silibinin groups. The middle and lower panels represent the relative expression levels of OGT and GAPDH, respectively, as observed by immunoblotting. In the two graphs on the right, the intensity of *O*-GlcNAcylation and OGT was determined by densitometry. Data are representative of three independent experiments. (**B**) Liver sections of ND, MCD/LPS, or MCD/LPS + silibinin-fed mice were subjected to immunohistochemical analysis with antibodies against OGT. ×200 magnification. (**C**) Relative expression levels of the proteins associated with the HBP pathway were determined by qRT-PCR analysis. Differential levels of *GFAT*, *GLUT4*, and *XBP1* mRNA are shown as fold-increase. Gene expression was normalized to 18 s RNA as an internal standard, and the data were presented as the mean value ± SE of seven samples. * *p* < 0.05 vs. ND group, and # *p* < 0.05 vs. MCD/LPS group.

For further analysis, we first investigated the changes in *O*-GlcNAcylation levels in steatohepatitis after treatment with silibinin. As shown in Figure 3A, MCD/LPS-fed mice were induced for not only *O*-GlcNAcylation but also for the upregulation of OGT expression. On the other hand, *O*-GlcNAcylation levels and OGT expression sharply decreased in silibinin-treated mice. The level of OGT expression was re-confirmed by immunohistochemistry (Figure 3B). Next, we examined the level of expression of HBP-related proteins through mRNA levels. In Figure 3C, the glutamine: fructose-6-phosphate aminotransferase (GFAT) levels were significantly upregulated in the MCD-fed mice, but dramatically suppressed by silibinin treatment. The expression of glucose transporter 4 (GLUT4) was increased in NASH, but decreased in silibinin-treated samples. Similar results were obtained for X-box binding protein 1 (XBP1), a transcription factor that regulates the immune system and ER stress response [23].

### 3.4. Silibinin Ameliorates Inflammation by Inhibiting NF-κB Signaling and Glcnacylation

**Figure 4:**
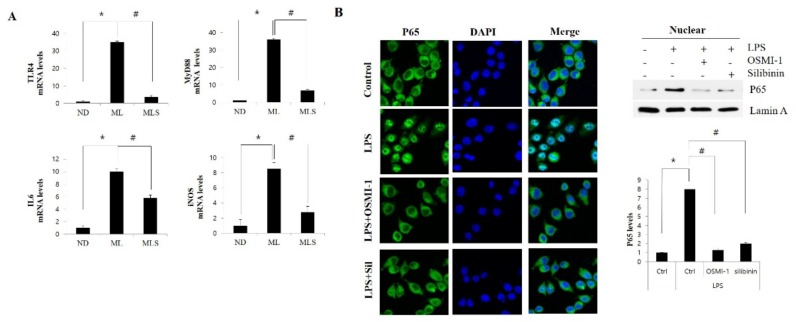

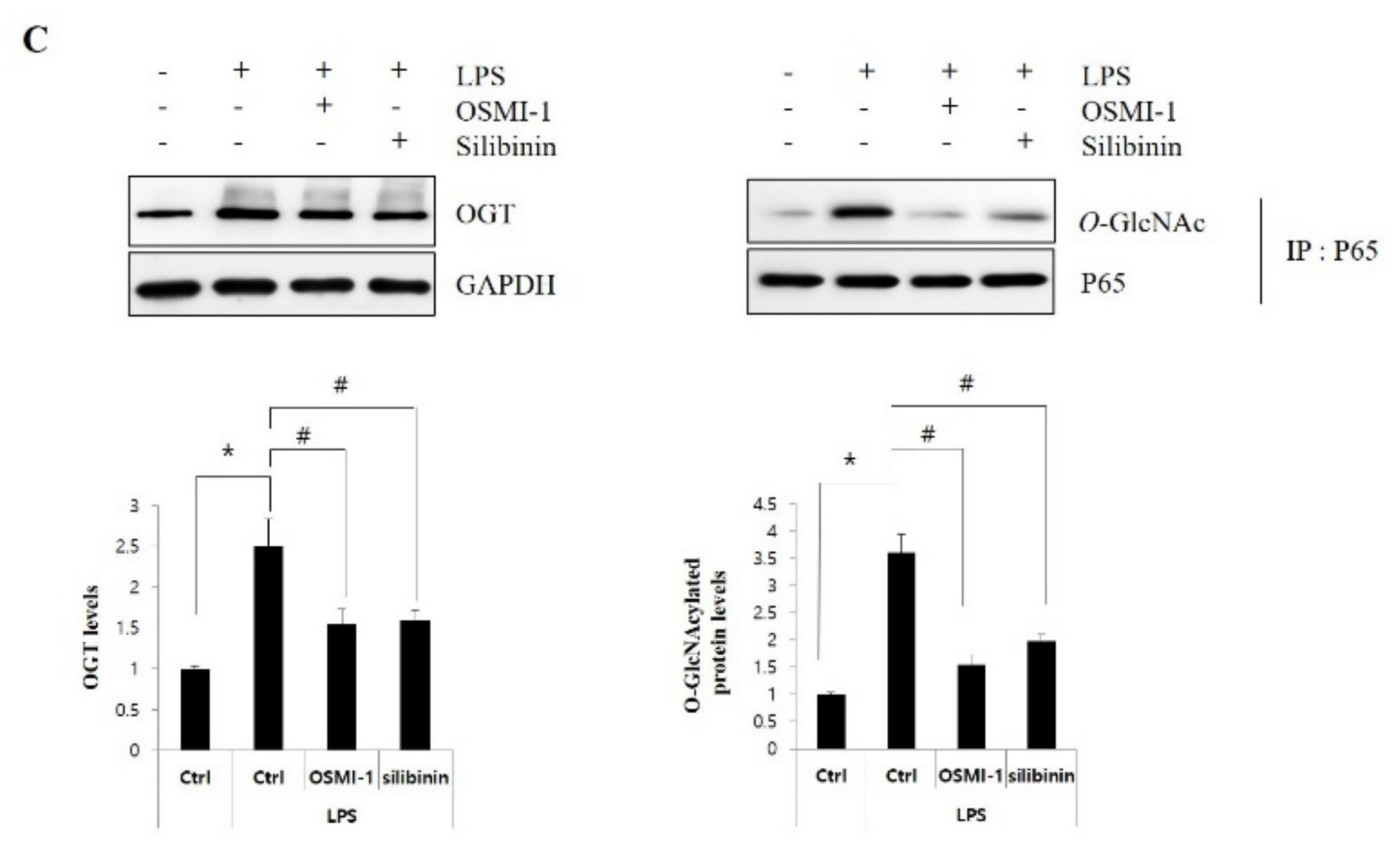
Silibinin inhibits the NF-κB-signaling pathway of NASH. (**A**) Liver tissue extracts were prepared from mice fed the ND, MCD/LPS, or MCD/LPS + silibinin diets. Hepatic mRNA levels of *TLR4*, myd88, and the NF-κB-mediated inflammatory cytokines IL-6 and iNOS were measured by qRT-PCR. Genes were normalized to *18s*RNA as an internal standard, and the data are expressed as fold-increase. * *p* < 0.05 vs. ND group, and # *p* < 0.05 vs. MCD/LPS group. All images shown are representative of three independent experiments. (**B**) Raw 264.7 cells were untreated or pretreated with OSMI-1 (50 µg/mL) or silibinin (100 μg/mL) for 30 min, and then stimulated with LPS (100 ng/mL) for 1 h. Immunofluorescent staining of NF-κB p65 or DAPI in raw 264.7 cells was performed with a kit, including ProLong^®^ Gold and SlowFade^®^ Gold Antifade. The right panel represents the relative nuclear expression levels of P65, respectively, as observed by immunoblotting. The secondary antibody was conjugated with a fluorescent green dye (Alexa Fluor 488). ×200 magnification. (**C**) Raw 264.7 cells were untreated or pretreated with OSMI-1 (50 µg/mL) or silibinin (100 μg/mL) for 30 min, and then stimulated with LPS (100 ng/mL) for 1 h. The upper left panel shows the extent of OGT expression by immunoblotting. Immunoprecipitation analysis using P65 antibody was performed, as shown in the upper right panel, followed by immunoblotting using GlcNAc antibody. Immunoprecipitation analysis was performed using whole cell lysate, and the GlcNAc levels were assessed using the same amount of precipitates. The lower two graphs show the relative amount of the above proteins as fold-increase. Data represent three independent experiments. * *p* < 0.05 vs. mock, and # *p* < 0.05 vs. LPS alone in each treatment.

LPS-mediated TLR4 activation is thought to play an essential role in MCD-feeding induced hepatitis. In order to explore LPS-mediated TLR4 signaling, real-time PCR analysis of the liver homogenates was performed. As shown in Figure 4A, TLR4 and the adapter MyD88 mRNA levels significantly increased in the LPS-injected MCD diet mice, but were restored to almost normal levels by treatment with silibinin. In addition, the gene expression of the NF-κB-dependent inflammatory cytokines IL6 and iNOS was also upregulated in LPS-injected MCD mice, but this increase was significantly inhibited by treatment with silibinin. In parallel with the liver tissue data, we also used macrophage raw 264.7 cells to investigate whether NF-κB p65 was translocated to the nucleus during TLR4 signaling. As shown in Figure 4B, immunofluorescence and immunoblotting analysis showed that NF-κB p65 accumulation in the nucleus was strongly induced when the cells were exposed to LPS for 60 min. However, the translocation of NF-κB to the nucleus was not observed after pretreatment with the OGT-specific inhibitor OSMI-1 or silibinin. In addition, we tested the role of *O*-GlcNAcylation in NF-κB signaling (Figure 4C). OGT expression levels in raw 264.7 cells were increased when treated with LPS alone, but this increase was considerably alleviated by further treatment with OSMI-1 or silibinin (left panel). Moreover, to confirm the involvement of *O*-GlcNAcylation in p65 activation, co-immunoprecipitation analysis with P65 antibody was performed, followed by immunoblotting using the *O*-GlcNAc antibody (right panel). Similarly, the degree of *O*-GlcNAcylation was increased by LPS treatment, which showed significant suppression after further treatment with OSMI-1 or silibinin.

## 4. Discussion

The cause of progression of steatohepatitis to NASH is still poorly understood. In order to gain more insight into the pathogenesis of NASH, we need further studies to identify highly specific proteins in steatohepatitis, and to characterize the metabolic pathways that might explain the onset of steatohepatitis. Here, for the first time, a differential proteomic approach was applied to study the influence of silibinin on the hepatic proteome in NASH mice. The results confirmed that 18 differentially expressed proteins were identified, which are related to metabolic enzymes, structural proteins, and the proteins involved in gene regulation and oxidative stress. All these proteins were differentially expressed in NASH, but they were significantly ameliorated by silibinin treatment. Interestingly, among the identified proteins, seven proteins, including CK8, CK18, PRX4, and protein disulfide isomerase, are known to undergo GlcNAcylation modification [24]. So far, no study has shown silibinin to inhibit the *O*-GlcNAcylation process to exert anti-inflammatory function in hepatitis. Thus, the aim of this work is twofold: to investigate the influence of silibinin on the liver proteome in NASH mice and to study how GlcNAcylation is associated with steatohepatitis. Consequently, we focused on these GlcNAcylated proteins and the proteins involved in HBP to assess and validate the positive correlation with the development of NASH.

High glucose and lipid levels are involved in the inflammatory process [25]. Hyperglycemia associated with a metabolic syndrome stimulates abnormally elevated *O*-GlcNAcylation [26,27]. Although HBP might function as a master regulator of inflammation signaling, the involvement of *O*-GlcNAcylation in inflammatory events is still obscure. Here, we investigated the changes in the *O*-GlcNAcylation levels as well as the effects of silibinin treatment on steatohepatitis (Figure 3). The results indicated that *O*-GlcNAcylation increased in NASH mice, and that HBP flux proteins, including OGT and GFAT, were also upregulated. Perhaps the increase in HBP flux and *O*-GlcNAcylation is linked with OGT expression. However, this upregulation was significantly alleviated by silibinin treatment. On the other hand, fructose-1,6-bisphosphatase (FBPase) expression was upregulated in the NASH model, but no change was observed in silibinin-treated mice, just like the ND mice (Table 1). This seems to explain the correlation between gluconeogenesis and *O*-GlcNAcylation. The MCD mice showed higher levels of gluconeogenesis, leading to an increase in the F6P levels by FBPase, which may lead to HBP flux, causing an upregulation of *O*-GlcNAcylation. Indeed, it has been demonstrated that FBPase is one of genes upregulated in the NASH by inflammation. Elevation of liver *O*-GlcNacylated proteins via an increased flux through the HBP appears to be the key linking the increase in liver FBPase to in NASH [28]. Conclusively, liver FBPase appears to regulate inflammation in NASH, because increased *O*-GlcNAcylation leads to cytokine expression. Thus, we propose that the upregulation of *O*-GlcNAcylation as sensed by HBP flux influences the regulation of inflammation.

Although the function is not yet clear, several cytoskeletal proteins undergo *O*-GlcNAc modification. The intermediate filament proteins CK8 and CK18, which are mainly found in epithelial cells, are classified as a neutral-basic type II keratin and a relatively acidic type I group, respectively [29,30]. In this study, we showed that the expression of CK8 and CK18 were significantly upregulated in NASH mice, while these effects were dramatically attenuated by silibinin treatment. More interestingly, we showed that the level of *O*-GlcNAcylation in these proteins was also upregulated in NASH mice, and suppressed by silibinin treatment. *O*-GlcNAc-modification of CK8 was first reported by Chou et al. [31], and CK18 is known to be modified by *O*-GlcNAc at a minimum of three sites (Ser-30/Ser-31/Ser-49) [32]. *O*-GlcNAcylation determines the solubility, filament organization, and stability of CK8/18 [33]. However, further investigation is needed to explore the role of *O*-GlcNAcylation in these cytoskeletal proteins in NASH.

Oxidative stress plays a pivotal role in the development of NASH. PRX4, an important antioxidant protein, is involved in the detoxification of peroxides, especially hydrogen peroxide and phospholipid hydroperoxide [34]. In this work, we showed that PRX4 was downregulated two-fold in silibinin-treated mice, compared to MCD/LPS mice. It also proved that the administration of silibinin leads to a substantial improvement in steatohepatitis, which seems to be caused by dysregulation of anti-oxidant defenses. *Prx4*-knockout mouse has recently been reported [35], which is characterized by the PRX4 elimination effect on spermatogenesis. Interestingly, PRX4 deficiency leads to an increase in cell death after oxidative stress [36]. Although PRX4 is also known to undergo *O*-GlcNAc modification, the *O*-GlcNAc site and its biological role are not yet known. In addition, PDIA3, also known as ERp57, mainly serves as a molecular chaperone belonging to the PDI family [37]. It is involved in several cellular functions, including protein folding in the endoplasmic reticulum and assembly of major histocompatibility complex I. It was reported that the expression of PDIA3 is highly upregulated in the liver of rats fed a high-fat diet [38]. However, the specific molecular mechanism of PDIA3 in NAFLD remains poorly understood. Our study showed that the expression of PDIA3 was upregulated in NASH mice, but was attenuated by silibinin treatment. We assumed that the *O*-GlcNAcylation of PDIA3 also participates in the pathogenesis of NASH through endoplasmic reticulum stress. Thus, the potential role of *O*-GlcNAcylation in the regulation of PDIA3 expression should be investigated in future studies.

Activation of the NF-κB pathway plays an important role in the response to acute inflammation injury in animal models [39,40]. Upon stimulation with LPS or free fatty acids, TLR4 activates MyD88-dependent signaling and induces NF-κB activation, leading to the production of the pro-inflammatory cytokine TNFα and the pro-inflammatory factor IL6 [41]. Furthermore, iNOS expression and nitric oxide (NO) production are upregulated in response to LPS, appearing to be mediated by NF-κB activation [42]. Here, we clearly demonstrated LPS-induced IL6 and iNOS expression through TLR4 upregulation in MCD-fed mice as well as the mitigating effects of silibinin treatment (Figure 4A). Moreover, upregulated *O*-GlcNAcylation is known to contribute to NF-κB activation [43]. The canonical NF-κB dimer constitutes the p65 and p50 subunits. *O*-GlcNAcylation of p65 is released from the sequestration by IκB, resulting in an increase in the nuclear translocation of NF-κB [44]. In this work, we also confirmed that silibinin inhibited TNF-α-induced activation of NF-κB pathway in macrophage raw 264.7 cells by blocking *O*-GlcNAcylation of NF-κB p65 (Figure 4B,C). The highly upregulated *O*-GlcNAcylation was involved in the activation of NF-κB pathway, while the inhibition of OGT by OGT inhibitor or silibinin treatment blocks the nuclear translocation of NF-κB p65. Thus, these results indicated that the involvement of silibinin in the anti-inflammatory effect is partially through the NF-κB-signaling pathways. Similarly, it has recently been reported that NF-κB forms a complex with OGT, and that *O*-GlcNAcylated NF-κB exerts an inhibitory effect [45]. Taken together, these data suggested that OGT expression and *O*-GlcNAcylation were upregulated in NASH mice, resulting in the translocation of NF-κB through *O*-GlcNAc modification of p65. Silibinin seems to inhibit the activity of NF-κB by blocking the *O*-GlcNAcylation process, which is essential for the translocation of NF-κB.

In summary, a differential proteomic approach allowed the identification of proteins whose expression is altered upon silibinin treatment in MCD-fed mice. Moreover, silibinin treatment results in a dramatic attenuation of upregulated GlcNAcylation. Here, we proposed that the anti-inflammatory effect of silibinin was mediated by blocking the NF-κB-signaling pathway through the inhibition of *O*-GlcNAcylation. To the best of our knowledge, the present study shows, for the first time, that silibinin inhibits *O*-GlcNAcylation to induce an inflammatory effect in NASH mice. The next step should be to assess the molecular mechanisms underlying the effect of silibinin on the *O*-GlcNAcylated candidate proteins identified in this work. GlcNAcylation studies provide a new perspective on the pathogenesis of steatohepatitis, and are likely to be used as potential biomarkers in the future.

## Figures and Tables

**Table 1:** Differentially expressed proteins list.

No ^a^	Identified Protein	Accession No.	MW (KDA)	PI	MOWSE SCORE	Coverage (%)	Fold Change (ND = 1)	
							ML	MLS
Metabolism								
1	Ornithine aminotransferase, mitochondrial	P29578	48.4	6.2	1.43 × 10^6^	33.7	0.5	0.8
2	Indolethylamine *N*-methyltransferase	P40936	29.5	6.0	282	28.8	0.2	0.8
3	Glutathione synthase	P51855	52.2	5.6	73,657	24.1	1.6	0.6
4	Fructose-1,6-bisphosphatase	Q9QXD6	36.9	6.1	1.23 × 10^6^	44.7	2	1.1
5	Annexin A5	P48036	35.8	4.8	1531	15.0	3	1.2
6	Zinc finger protein 330	Q922H9	35.6	5.8	50.9	17.1	0.3	1
**Structural**								
7	Keratin, type II cytoskeletal 8 *	P11679	54.6	5.7	20,152	21.0	1.4	0.8
8	Keratin, type II cytoskeletal 18 *	P05784	47.5	5.2	3.72 × 10^7^	36.6	4	1.2
9	Protein disulfide-isomerase A3 *	P22273	56.6	5.9	66,094	34.3	1.5	1
10	Actin, cytoplasmic 1 *	P60710	41.7	5.3	5421	21.1	5	2.5
11	Rho GDP-dissociation inhibitor 1 *	Q99PT1	23.4	5.1	1960	59.3	2.5	1.4
12	Tropomyosin alpha-1 chain	P58771	32.7	4.7	32.5	9.9	3	1
**Genomic**								
13	Transcription factor E2F6	Q61502	36.6	4.9	110	11.0	0.4	0.7
14	40s ribosomal protein SA *	P14206	32.8	4.8	21,692	40.3	1.7	1
15	Protein NDRG2	Q9QYG0	40.8	5.2	31,743	29.4	0.2	0.9
16	Proliferating cell nuclear antigen	P17918	28.8	4.7	100	11.5	1.5	0.9
**Oxidative stress**								
17	Peroxiredoxin-4 *	Q08807	31.0	6.7	4.96 × 10^6^	48.5	5	2
18	Regucalcin	Q64374	33.4	5.2	480,847	42.5	0.3	1.2

^a^ Protein ID numbers correspond to the spot numbers on the 2-DE gel in Figure S2. *: indicates O-GlcNAc modifiable protein.

## Supplementary Materials

The following are available online at http://www.mdpi.com/1422-0067/19/8/2165/s1.
