# Supplementary material for: Silibinin Ameliorates O-GlcNAcylation and Inflammation in a Mouse Model of Nonalcoholic Steatohepatitis
Source: Int J Mol Sci. 2018 Jul 24;19(8):2165. doi: 10.3390/ijms19082165 (PMC6121629; doi:10.3390/ijms19082165)
Supplement: Supplementary file 1 [file ijms-19-02165-s001.pdf]

Supplementary Materials

Silibinin Ameliorates *O*-GlcNAcylation and Inflammation in a Mouse Model of Nonalcoholic Steatohepatitis

Su Jin Lee, Min Jung Nam, Da Eun Lee, Jeen-Woo Park, Beom Sik Kang, Dong-Seok Lee, Hyun-Shik Lee and Oh-Shin Kwon *

School of Life Science, College of Natural Science, Kyungpook National University, Daegu 41566, Korea; neojove79@naver.com (S.J.L.); nmj0604@naver.com (M.J.N.); Starkr0@hanmail.net (D.E.L.); parkjw@knu.ac.kr (J.-W.P.); bskang2@knu.ac.kr (B.S.K.); lee1@knu.ac.kr (D.-S.L.); leeh@knu.ac.kr (H.-S.L.)
* Correspondence: oskwon@knu.ac.kr; Tel.: +82-53-950-6356; Fax: +82-53-943-2762

Received: 1 June 2018; Accepted: 19 July 2018; Published: 24 July 2018

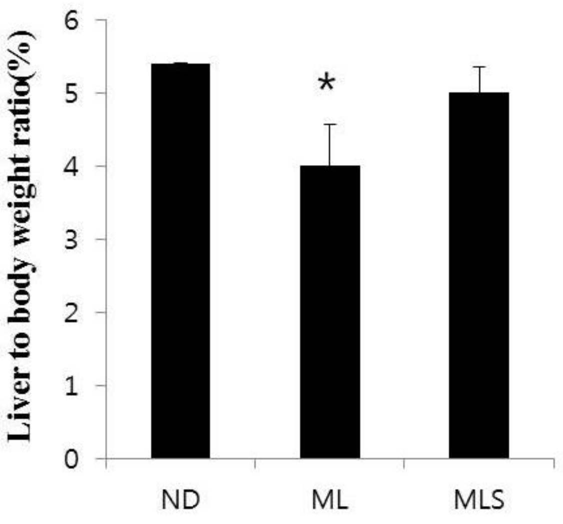

Figure S1. MCD+LPS diet-induced liver steatohepatitis. MCD diet affected body weight and liver weights levels of ND, MCD/LPS diet, or MCD/LPS diet + silibinin for three weeks.

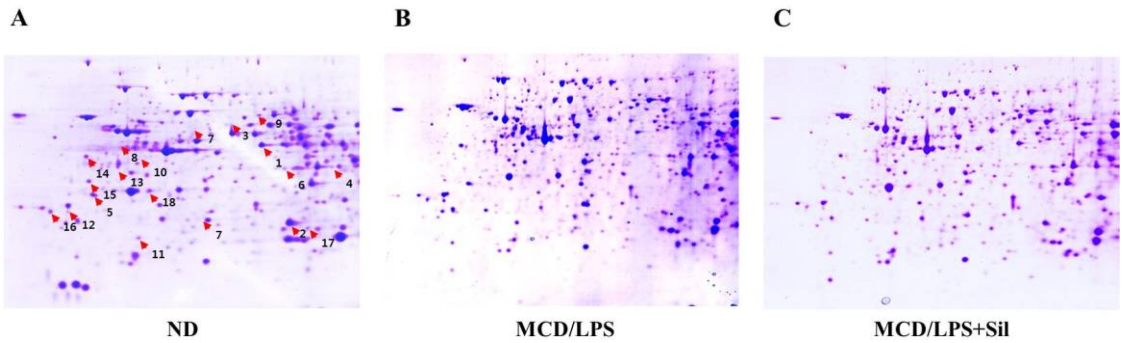

Figure S2. Protein expression map of mouse liver. Coomassie Blue-stained 2DE gel shows the proteins derived from mice fed ND, MCD/LPS, or MCD/LPS + silibinin-treated diet. The proteins from the mouse

liver were loaded on a 24 IPG strip (pH 4–7) and subjected to SDS-PAGE (12%). The protein spots significantly affected by different diets are indicated by arrows. The numbers on the gel correspond to the spot numbers in Table 1.

Table S1. Primers used for real-time PCR.

Primer name	Sequences
18S rRNA	(F)5'-GTA ACC CGT TGA ACC CCA TT-3'
	(R)5'-CCA TCC AAT CGG TAG TAG CG-3'
TNFα	(F)5'-CAC CAC CAT CAA GGA CTC AA-3'
	(R)5'-AGG CAA CCT GAC CAC TCT CC-3'
Myd88	(F)5'-AGA ACA GAC AGA CTA TCG GCT-3'
	(R)5'-CGG CGA CAC CTT TTC TCA AT-3'
TGFβ1	(F)5'-ATT CCT GGC GTT ACC TTG-3'
	(R)5'-CTG TAT TCC GTC TCC TTG GTT-3'
IL-6	(F)5'-GAC AAC TTT GGC ATT GTG G-3'
	(R)5'-ATG CAG GGA TGA TGT TCT G-3'
TLR4	(F)5'-ACC TCT GCC TTC ACT ACA GA-3'
	(R)5'-AGG GAC TTC TCA ACC TTC TC-3'
iNOS	(F)5'-CTG CAG CAC TTG GAT CAG GAA CCT G-3'
	(R)5'-GGG AGT AGC CTG TGT GCA CCT GGA A-3'
XBP1s	(F)5'-AAG AAC ACG CTT GGG AAT GG-3'
	(R)5'-ACT CCC CTT GGC CTC CAC-3'
GFAT1	(F)5'-TAA GGA GAT CCA GCG GTG TC-3'
	(R)5'-CAG CTG TCT CGC CTG ATT GA-3'
GLUT4	(F)5'-GAT TCT GCT GCC CTT CTG TC-3'
	(R)5'-ATT GGA CGC TCT CTC TCC AA-3'
